# Genome-Wide Association Study of Brown Rot (*Monilinia* spp.) Tolerance in Peach

**DOI:** 10.3389/fpls.2021.635914

**Published:** 2021-03-09

**Authors:** Wanfang Fu, Cassia da Silva Linge, Ksenija Gasic

**Affiliations:** Department of Plant and Environmental Sciences, Clemson University, Clemson, SC, United States

**Keywords:** disease resistance, association mapping, fruit breeding, candidate gene analyses, *Rosaceae*, *Prunus*

## Abstract

Brown rot, caused by *Monilinia* spp., is one of the most important diseases on stone fruit worldwide. Severe yield loss can be caused by pre- and post-harvest fruit decay. Although some degree of tolerance has been reported in peach and almond, the genetic resistance in peach cultivars is still lacking. To date, only few genomic regions associated with brown rot response in fruit skin and flesh have been detected in peach. Previous studies suggested brown rot tolerance in peach being a polygenic quantitative trait. More information is needed to uncover the genetics behind brown rot tolerance in peach. To identify the genomic regions in peach associated with this trait, 26 cultivars and progeny from 9 crosses with ‘Bolinha’ sources of tolerance, were phenotyped across two seasons (2015 and 2016) for brown rot disease severity index in wounded and non-wounded fruits and genotyped using a newly developed 9+9K peach SNP array. Genome wide association study using single- and multi-locus methods by GAPIT version 3, mrMLM 4.0, GAPIT and G Model, revealed 14 reliable SNPs significantly associated with brown rot infection responses in peach skin (10) and flesh (4) across whole genome except for chromosome 3. Candidate gene analysis within the haplotype regions of the detected markers identified 25 predicted genes associated with pathogen infection response/resistance. Results presented here facilitate further understanding of genetics behind brown rot tolerance in peach and provide an important foundation for DNA-assisted breeding.

## Introduction

Brown rot, caused by *Monilinia* spp., is one of the most economically important diseases in stone fruits (Van Leeuwen et al., 2002). This worldwide disease favors warm and humid growing conditions (Bernat et al., 2017) and can lead to severe yield losses in both pre- and post-harvest stages. In the United States, *M. fructicola* is the predominate species that causes brown rot in peach while *M. laxa* and *M. fructigena* are causative fungi in Europe (Byrde and Willetts, 1977; Lino et al., 2016). As an ascomycete, *Monilinia* can affect the peaches at two growth stages: blossom and twig blight caused by ascospores and conidia in the early spring and pre- and post-harvest fruit decay caused by conidia infection in the late spring and summer (Zehr, 1982; Tate and Wood, 2000; Luo et al., 2005; Lino et al., 2016).

Currently, in the commercial peach production, brown rot is primarily controlled by routine fungicide sprays at the bloom and during pre-harvest (Sharma, 2003; Rungjindamai et al., 2014). Since *Monilinia* infection can be highly impacted by the environment, brown rot disease severity in the orchard can vary in different years, and in a season favorable for infection the number of fungicide applications can be extremely high (Pacheco et al., 2014; Bernat et al., 2017; Garcia-Benitez et al., 2017). Consequently, consumer’s awareness of potential health and environmental problems caused by agricultural chemical residues have increased (Byrne, 2001, 2005). In addition, *Monilinia* strains with resistance to several fungicide classes have been reported (Luo and Schnabel, 2008; Chen et al., 2013, 2015), suggesting chemical control might become less efficient. Although, biological control using microbial agents is underexplored, this method is still not feasible for practical applications (Martínez-García et al., 2013). Therefore, host-derived resistance/tolerance seems to be the most effective strategy to reduce the chemical input, as well as to facilitate sustainable and eco-friendly cultivation practices. Some degree of tolerance has been observed in Brazilian landrace cultivar Bolinha (Feliciano et al., 1987) and the advanced selections with almond background developed by University of California, Davis (Martínez-García et al., 2013). However, most of the commercial cultivars are susceptible to brown rot. Thus, developing cultivars that combine disease tolerance/resistance with superior fruit quality and productivity is an important goal in most peach breeding programs.

The mechanisms of brown rot tolerance remain unclear. Fruit epidermis is the first barrier for preventing *Monilinia* infection as studies have shown that fruits with multiple layers of epicuticular waxes and thicker cuticle tend to be more resistant to the disease (Gradziel et al., 2003; de Oliveira Lino et al., 2015). In addition, the natural openings, such as stomata, can serve as “channels” to facilitate *Monilinia* infection in peach (de Oliveira Lino et al., 2015). High concentration of phenolic compounds (chlorogenic acid, caffeic acid), chlorophyll and pectin were indicated as potential sources of resistance in the peach epidermis (Gradziel et al., 1997, 2003; Bostock et al., 1999); but epidermal barrier is vulnerable to mechanical injury and insect damage. Flesh tolerance due to high levels of phenols (chlorogenic and neochlorogenic acid) and polyphenol oxidase enzyme activity (correlated with high enzyme browning) has been observed, however, increased levels of these compounds cause flesh browning which is commercially undesirable (Gradziel and Wang, 1993; Gradziel et al., 1997; Gradziel et al., 2003; Villarino et al., 2011). Ripening process changes the structure and biochemical composition of peach fruit making it increasingly susceptible to brown rot and enabling activation of latent infections (Gradziel, 1994; Mari et al., 2003; Villarino et al., 2011; Guidarelli et al., 2014; Garcia-Benitez et al., 2017).

Previous studies revealed polygenetic and quantitative nature of brown rot resistance/tolerance in peach (Martínez-García et al., 2013; Pacheco et al., 2014; Baró-Montel et al., 2019). QTL regions associated with brown rot tolerance have been reported in both peach (Pacheco et al., 2014) and peach × almond hybrids (Martínez-García et al., 2013; Baró-Montel et al., 2019). Pacheco et al. (2014), mapped QTLs associated with skin resistance on linkage group (LG) 2, and flesh resistance on LG3 using an F1 progeny from the cross between commercial cultivars Contender (moderate tolerance) × Elegant Lady (susceptible). Martínez-García et al. (2013) detected two QTLs on LG1 associated with brown rot resistance/tolerance in peach fruit by analyzing the wounded fruits (skin was breached) of the F1 progeny derived from an interspecific cross between peach cultivar Dr. Davis and an almond × peach F_2_BC_2_ introgression line F8, 1–42. Two candidate genes, *ppa011763m* and *ppa026453m*, were indicated as responsible for *M. fructicola* recognition in peach. Recently, Baró-Montel et al. (2019) evaluated an interspecific BC1 population between ‘Texas’ (almond) and ‘Earlygold’ (peach) and mapped 12 QTL regions on several LGs (except LG1 and 3) involved in brown rot tolerance in skin/flesh. Despite the importance of the Brazilian cultivar ‘Bolinha’ (Feliciano et al., 1987) as a source of resistance to brown rot, no QTL mapping studies have been performed using this cultivar as a parent in biparental crosses.

Linkage mapping, used in previous studies, was restricted to genetic diversity present in the parents of segregating populations (Zhu et al., 2008). Association mapping is an alternative approach that exploits linkage disequilibrium and historical recombination in a diverse germplasm (Singh and Singh, 2015). There are several models available for use in association mapping. Population structure (Q) and kinship (K) matrix as cofounding factors are commonly used to improve the estimate of trait-marker association for each tested marker (Yu et al., 2006). Common genome-wide association study (GWAS) methods based on single-locus models, such as mixed linear model (MLM) and general linear model (GLM), require Bonferroni correction for multiple tests to control false positive rate. However, this type of correction is typically very conservative (Wang et al., 2016) and results in many important loci associated with the target trait being excluded because of the stringent criteria (Zhang et al., 2019). To overcome this issue, multi-locus strategies have been developed as alternatives to single locus methods (Wang et al., 2016). Instead of testing each single marker in a one-dimensional genome scan, multi-locus (GWAS) methods estimate the effect of all markers simultaneously with a multi-dimensional genome scan, therefore can represent appropriate genetic models for complex trait (Wang et al., 2016; Zhang et al., 2019). The multi-locus methods showed higher quantitative trait nucleotide (QTN) detection power without compromising the false positive rate (Zhang et al., 2019).

GAPIT is an R package widely used for GWAS analysis, and the recently updated version 3 (Wang and Zhang, 2020) implemented 3 multi-locus methods, including Multiple Loci Mixed Model (MLMM) (Segura et al., 2012), Fixed and random model Circulating Probability Unification (FarmCPU; Liu et al., 2016), and Bayesian-information and Linkage-disequilibrium Iteratively Nested Keyway (BLINK) (Huang et al., 2019), in addition to the previously implemented single-locus methods.

Recently, an R package, mrMLM v4.0 (Zhang et al., 2020), that integrates six multi-locus methods including mrMLM (Wang et al., 2016), FASTmrMLM (Tamba and Zhang, 2018), FASTmrEMMA (Wen et al., 2018), pLARmEB (Zhang et al., 2017), pKWmEB (Ren et al., 2018) and ISIS EM-BLASSO (Tamba et al., 2017) was developed for multi-locus GWAS. The putatively associated markers are first selected by various algorithms, and later are put in one model for estimating the effects by empirical Bayes and all the non-zero effects are further identified by likelihood ratio test for the true QTNs (Zhang et al., 2020).

Another multi-locus-based method, G model developed by Bernardo (2013), uses the estimated background marker effects directly to reflect polygenic background effects, unlike the QK model which exploits the polygenic background effects by estimating the kinship. The G model showed superior performance for controlling the false positive errors and detecting true QTNs in comparison with QK model (Bernardo, 2013). GWAS in peach have been used for mapping fruit quality (Dhanapal and Crisosto, 2013; Micheletti et al., 2015; Font I Forcada et al., 2019; Tan et al., 2019), disease resistance (Cirilli et al., 2017) and agronomic traits (Cao et al., 2016) taking advantage of a high throughput genotyping via IPSC peach 9K SNP array v1 (Verde et al., 2012) and next generation sequencing. To our knowledge this is the first report on understanding peach fruit response to brown rot infection using association mapping. In this study we applied several models, including both single- and multi-locus methods, to simultaneously detect markers associated with brown rot tolerance in peach and identified candidate genes associated with peach responses to *Monilinia* infection. Results presented here contribute to understanding of genetics behind brown rot tolerance in peach and provide an important foundation for genome-assisted breeding.

## Materials and Methods

### Plant Material

A total of 26 cultivars/advanced selections and the 138 progeny from 9 breeding families (Supplementary Table 1) with ‘Bolinha’ source of resistance were included in this study. The plant material is maintained at the Clemson University Musser Fruit Research Center, Oconee county in Seneca, South Carolina (Latitude: 34.639038, Longitude: -82.935244, Altitude 210msl), under warm, humid, temperate climate and standard commercial practices for irrigation, fertilization and pest and disease control. The trees were at least 5 years old, grafted on Guardian^®^ rootstock, and either planted at 4 × 6m and trained to open center or planted at 1.5 × 4m and trained to perpendicular V.

### Phenotyping

Forty fruits per accession were bagged in the spring after the first fungicide application to prevent further fungicide residue deposit. The bagged fruit was harvested at the commercial maturity and 20 fruits of similar maturity, Index of Absorbance Difference (I_AD_) 0.6–0.8, were selected for brown rot inoculation. For each available accession in the pedigree, parallel inoculation was conducted on 10 fruits each under both wounded (skin was breached) and non-wounded treatment (Martínez-García et al., 2013) using *M. fructicola* isolates KH-13 as inoculum. Lesion diameters (mm) were recorded for each individual and disease severity index (DSI) was calculated as the product of average lesion diameter × disease incidence (proportion of lesions greater than 3mm) following protocol described in Fu et al. (2018). Statistical analyses were carried out by SPSS Statistics v. 27 (IBM^®^). Frequency histograms were generated to detect the distributions of the phenotypic data. Normality of the datasets were tested by Shapiro–Wilk test with a *p*-value threshold of 0.05. Correlation analysis between the different treatment and year was performed using Spearman’s rank correlation coefficients (R) at *p* < 0.05.

Non-wounded and W DSI were fitted with to a linear mixed model using R package ‘lme4’ (Bates et al., 2015), in which the year and block were selected as random effects:

$$\begin{array}{rc} \mathrm{Trait}= & \left(1|\mathrm{Accession}\right)+\left(1|\mathrm{Year}\right)+\left(1|\mathrm{Block}\right)+\\ & \left(1|\mathrm{Accession}:\mathrm{Year}\right)+\left(1|\mathrm{Accession}:\mathrm{Block}\right) \end{array}$$

The broad sense heritability was estimated as the ratio of total genetic variance to total phenotypic variance.

### Genotyping

DNA was extracted from young leaves following the Dellaporta et al. (1983) protocol. DNA quantification was performed using QuantiFluor^®^ dsDNA (Promega) adjusted to 50 ng/μl and submitted to the Research Technology Support Facility at Michigan State University (East Lansing, MI, United States). Genotyping was performed using newly developed 9+9K peach SNP array (Gasic et al., 2019). The newly developed peach SNP array contains SNPs from the IPSC peach 9K SNP array v1 (Verde et al., 2012) with addition of 7894 SNPs (www.rosbreed.org).

SNP genotypes were scored with the Genotyping Module of GenomeStudio Data Analysis software (Illumina Inc.). SNP filtering was done using ASSIsT v1.02 (Di Guardo et al., 2015) under the germplasm panel with default parameters to remove the null alleles and the monomorphic markers. The polymorphic SNPs which passed the filtration step and had missing data (No Call) lower than 10% were included. Markers with minor allele frequency (MAF) higher than 0.05 (Vanderzande et al., 2019) were selected by PLINK 1.9 (Chang et al., 2015) for further analysis.

### Population Structure Analysis and Genome Wide Association Study (GWAS)

To investigate the population structure of the GWAS panel, we used a Bayesian clustering in fastSTRUCTURE (Raj et al., 2014). A number of clusters (K) ranging from 1 to 10 were tested using the default priors. The chooseK.py script in fastStructure was used to estimate the reasonable range of K for the appropriate model complexity. The admixture proportions of each genotype were visualized by DISTRUCT plots (Rosenberg, 2004).

The GWAS was performed using both single- and multi-locus models, by GAPIT version 3 (Wang and Zhang, 2020), mrMLM 4.0 (Zhang et al., 2020), FarmCPU (Liu et al., 2016) and G model (Bernardo, 2013; Table 1). G model was carried out using GModel2 software^1^. GModel2 requires non-missing values in the input files, therefore individuals with genotyping rate lower than 90% and makers with missing data were excluded from the analysis. Moreover, linkage disequilibrium (LD) pruning was applied using PLINK 1.9 (Chang et al., 2015) for excluding the SNPs within the same chromosome that had *r*^2^ > 0.85. A *p*-value threshold of 1E-07 (highly significant and far surpassing the Bonferroni correction) was considered as a cutoff to call the significant trait-marker associations.

**TABLE 1:** Models used for GWAS analysis.

Package/Software	Model name	Model type	Covariates	Significant critical value
GAPIT Version 3	MLM	Single-locus	Q+K	*p*-value ≤ 0.05/n
	GLM		Q	
	CMLM		Q+K	
	SUPER		Q+K	
	MLMM	Multi-locus	S+Q+K	
	BLINK		S	
FarmCPU	FarmCPU	Multi-locus	S+K	*p*-value ≤ 0.01/n
mrMLM 4.0	mrMLM	Multi-locus	S+Q+K	LOD score ≥ 3
	FASTmrMLM			
	FASTmrEMMA			
	pLARmEB			
	pKWmEB			
	ISIS EM-BLASSO			
GModel2	G Model	Multi-locus	G	*p*-value ≤ 0.0000001

*Q: population structure. K: kinship. S: pseudo QTNs. G: genome wide markers. n: number of markers included in test.*

All the other GWAS approaches were performed for the same individuals used in G model analysis without excluding the markers with missing data and applying LD pruning, and analyses were performed in R V4.0.1 with the corresponding packages. Six different models, including four single-locus models (MLM, GLM, CMLM and SUPER) and two multi-locus models (BLINK and MLMM), were applied using GAPIT version 3 (Wang and Zhang, 2020). The six multi-locus GWAS methods (mrMLM, FASTmrMLM, FASTmrEMMA, pLARmEB, pKWmEB, and ISIS EM-BLASSO) from mrMLM 4.0 (Zhang et al., 2020) were also used in this study. The Q matrices obtained from fastStructure were included as covariates in GAPIT, mrMLM and FarmCPU analysis, and for both GAPIT version 3 and mrMLM 4.0, all parameters in GWAS were set at default values, as the significant marker-trait associations were determined by the p-values ≤ 0.05 with Bonferroni correction (GAPIT Version 3) or LOD score ≥ 3 (mrMLM 4.0). Concerning FarmCPU, the default *p*-value threshold of 0.01, to select the pesudo-QTNs into the model for the first iteration with Bonferroni correction, can be overly strict when the genotypic markers have large LD. Therefore, we set this threshold to 0.05. Markers were defined as being significantly associated with the traits when the p-values were lower than 0.01 with Bonferroni correction.

In this study, the reliable marker-trait associations were considered when markers were repeatedly detected in at least two methods and/or two datasets using GAPIT version 3, mrMLM 4.0 and FarmCPU; or when markers detected in any of the three R packages shared the same haploblock with the markers detected in the GModel2.

Statistical difference among different genotype of significant associated marker were detected by performing ANOVA and Dunnett’s T3 test in SPSS v.27 (IBM^®^) at significance of *p* < 0.05.

### Candidate Gene Analysis

Haploblocking of the peach genome was performed using flag ‘–blocks-max-kb’ in PLINK 1.9 (Chang et al., 2015) within 1 Mb surrounding significant SNP. Only the haploblock regions detected with reliable marker-trait associations were used for candidate gene analysis. From the *Prunus persica* Whole Genome v2.0 Assembly and Annotation v2.1 (GDR)^2^ (Verde et al., 2017; Jung et al., 2019), a systematic search was conducted to compile the predicted candidate genes associated with disease resistance mechanisms. The coding sequences of these genes were further blasted against NCBI nr^2^ using blastn to compare with peach. Only the sequences with query cover more than 0.8 and E-value lower than 1E-50 were considered.

## Results

### Phenotypic Performance of GWAS Panel

**FIGURE 1:**
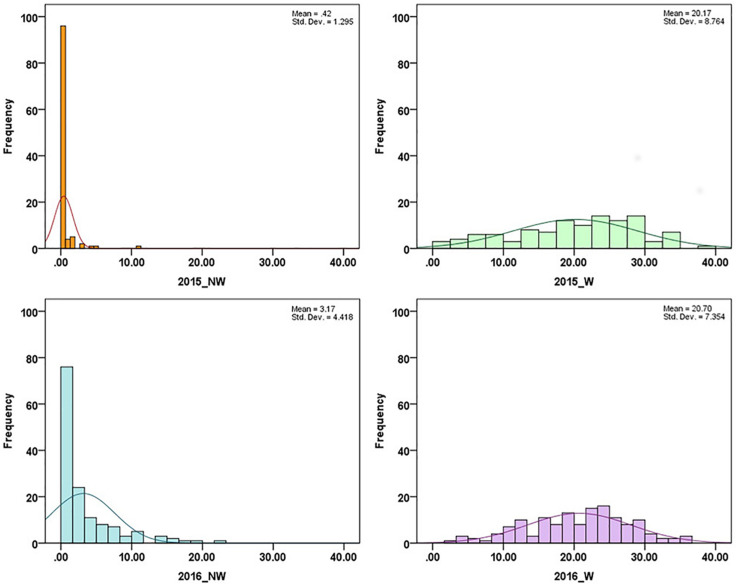
Disease severity index (DSI) distribution in association panels under non-wounded (NW) and wounded (W) treatment in 2015 and 2016.

In 2015 and 2016, 26 cultivars/advanced selections and 138 progeny from nine crosses (Supplementary Table 1) were evaluated for fruit responses to brown rot inoculation in wounded (W) and non-wounded (NW) fruits. Mean disease severity index (DSI) for 2015 NW, 2016 NW, 2015 W and 2016 W were 0.42, 3.17, 20.16 (moderate tolerance) and 20.70 (moderate tolerance), respectively. The frequency distributions of DSI under each treatment in the two different seasons are illustrated in Figure 1. DSI under NW treatment showed non-normal distribution. Since most of the accessions showed no signs of infection or very low level of infection after the inoculation, the DSI distribution of NW treatment was skewed toward low DSI. For W treatment, similar distribution pattern was observed in two years. However, Shapiro–Wilk test, showed only DSI of 2016 W dataset as normally distributed (*p* = 0.18). None of the accessions showed complete flesh resistance. Details about the phenotypic performance of the cultivars/advanced selections and crosses have been described in Fu et al. (2018). Spearman’s rank correlations of the phenotypic performance were shown in Table 2. W DSI (*r* = 0.507, *p* < 0.01) showed better correlation than NW DSI (*r* = 0.349, *p* < 0.05) between two different years. Considering the same year, NW DSI was positively correlated to W DSI in both 2015 (*r* = 0.429, *p* < 0.05) and 2016 (*r* = 0.648, *p* < 0.01). Estimated broad sense heritability (H^2^) for NW and W DSI were 0.18 and 0.35, respectively.

**TABLE 2:** Spearman’s rank correlation coefficients for Disease Severity Index (DSI) between years and treatments.

	2015_NW	2015_W	2016_NW	2016_W
2015_NW	1	0.429*	0.349**	0.276*
2015_W		1	0.344*	0.507**
2016_NW			1	0.648**
2016_W				1

*. Correlation is significant at the 0.05 level (2-tailed). **. Correlation is significant at the 0.01 level (2-tailed).

### Genotyping

Out of 16,038 SNPs on 9+9K array, 9,282 were polymorphic in the association panel. SNPs with MAF < 0.05 were further removed leaving a total of 8,365 SNPs for the subsequent analysis. The SNP dataset corresponded to an average of 36.4 SNPs/Mb (considering the total peach genome size of 230 Mb).

### Datasets

**FIGURE 2:**
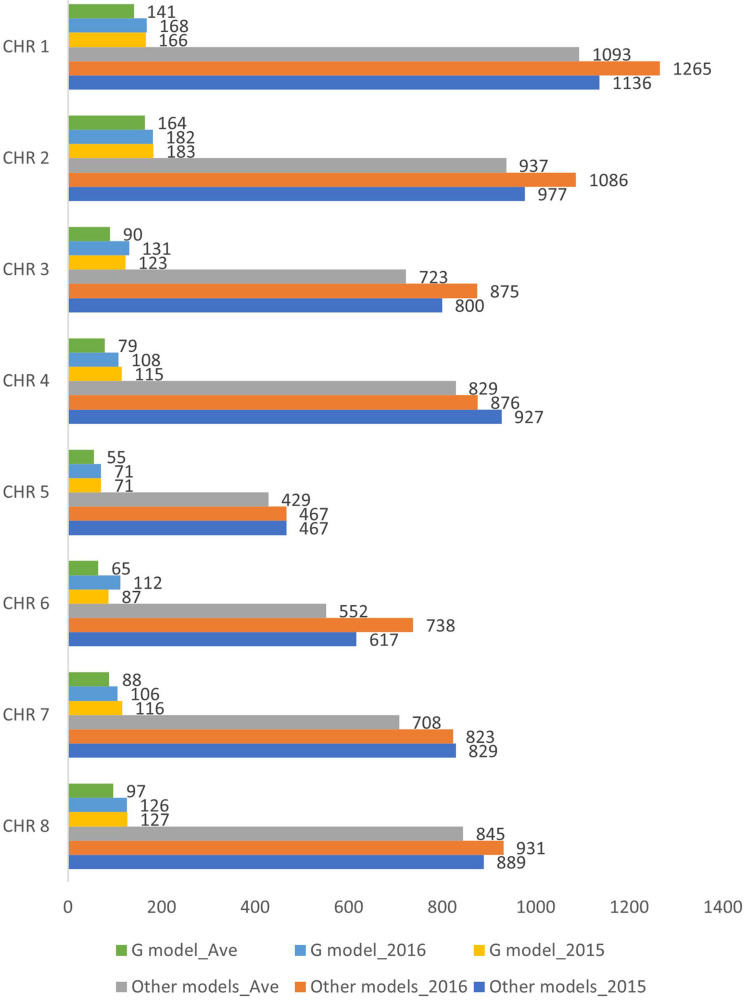
Number of SNPs included in the genome wide association analysis for the six datasets (G model_2015, G model_2016 G model_Ave, Other models_2015, Other models_2016 and Other models_Ave). CHR, chromosome.

After filtering for 90% genotyping rate, a total of 145 individuals (Supplementary Table 1) were included in the association study. Three different phenotypic datasets were used for the marker-trait association analyses: individuals evaluated under wounded (W) and non-wounded (NW) treatment in separate years (2015 and 2016), as well using the two-year DSI average for individuals that were phenotyped in both years (Ave). The final number of individuals included in 2015, 2016 and Ave were 103, 128 and 86, respectively. The G model and other single-/multi-locus models (Table 1) were used to carry out the association analysis. For each method, three different genotypic datasets were generated corresponding to the 3 phenotypic datasets. After removing the markers with missing value and LD pruning, the final numbers of SNPs included for 2015, 2016 and Ave G model datasets were 988, 1004 and 779, respectively. For other models, 6642, 7061 and 6116 SNPs were included in the corresponding 2015, 2016 and Ave datasets (Figure 2).

### Population Structure and Genome Wide Association Study

Population structure analysis with fastStructure suggested K number between 2 and 7 for 2015 dataset, 2 and 8 for 2016 dataset, and 7 and 8 for Ave dataset. When compared to the pedigree, *K* = 7 best explained the population structure for all three datasets (Supplementary Figure 1).

Genome wide association study via GAPIT revealed 7 different marker-trait associations, after Bonferroni correction (*p*-value ≤ 0.05), for DSI in both W (5) and NW (2) peach fruit on chromosomes (Chr) 2, 4, 6 and 7, according to all three datasets (2015, 2016, Ave) (Supplementary Table 2 and Supplementary Figure 3). GWAS using the six-multi-locus methods in mrMLM 4.0 revealed a total of 20 marker-trait associations (LOD ≥ 3) on Chr 2, 4, 5, 6, 7, and 8 (Supplementary Figure 5) for both W and NW DSI in all three data sets, with marker effect ranging from −7.52 to 3.21 (Supplementary Table 2).

Using FarmCPU, 12 markers significantly associated with skin (3) and flesh (9) tolerance were detected (Supplementary Figure 4). Significant markers, identified with the Bonferroni threshold of 0.01, with marker effect ranging from −7.91 to 7.34, were detected on Chr 4, 6, 7, and 8 (Supplementary Table 2).

Genome wide association study via GModel2 revealed significant marker-trait associations for DSI in both wounded and non-wounded peach fruits throughout the whole genome. In total, 32 SNPs (*p* < 0.0000001) were significantly associated with W (12) and/or NW (21) fruit responses to *Monilinia* infection according to the three different datasets (2015, 2016 and Ave), with the marker effect ranging from −5.42 to 5.52 (Supplementary Table 2). No SNPs associated with flesh tolerance were detected with 2015 and Ave datasets. One SNP on Chr 1, Peach_AO_0100564, was associated with peach fruit skin tolerance in both 2016 and Ave datasets. Another SNP on Chr 4, was associated with peach fruit skin and flesh tolerance according to the 2016 dataset.

**FIGURE 3:**
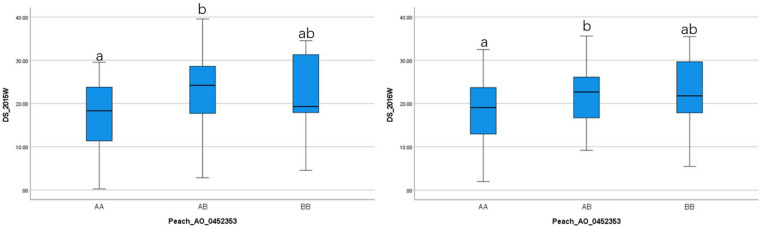
Comparison of the phenotypic performance for difference genotypes of Peach_AO_0462363 in two different seasons (2015 and 2016) in wounded (W) fruits.

To ensure reliable results, we considered the real marker-trait associations when the following criteria were met: the marker was detected in at least two methods and/or two datasets using GAPIT version 3, mrMLM 4.0, FarmCPU and GModel2; or markers detected in any of the models shared the same haploblock to the markers detected in GModel2. Using this criteria we detected 14 reliable marker-trait associations on all chromosomes, except Chr 3, with 10 of them associated with NW DSI and 4 of them associated with W DSI (Table 3). The phenotypic variance (*r*^2^) explained by the markers associated with NW and W DSI ranged from 0 to 22.76 and 4.82 to 40.45, respectively. Only one marker, Peach_AO_0243498, was detected by both single-locus (SUPER) and multi-locus models (Blink; pLARmEB; ISIS EM-BLASSO). Two markers, Peach_AO_0602163 and Peach_AO_0711526, were detected by more than 4 models at the same time, while five markers on Chr 1, 4, 6, 7 were detected with different datasets with same and/or different methods. According to the phenotypic variance explained by each significant marker, Peach_AO_0452353, related to flesh resistance on Chr 4 showed the highest *r*^2^ (31.44 – 40.45), thus we compared the phenotypic performance between different genotypes for this marker. Comparison of the phenotypic performance between different genotypes showed significant differences in both seasons (2015_W, 0.002; 2016_W, 0.020) with individuals homozygous for both alleles showing lower DSI than heterozygous individuals, and allele A having the lowest DSI (Figure 3).

**TABLE 3:** Significant markers associated with disease severity index (DSI) in wounded (W) and non-wounded (NW) treatments either repeatedly detected in at least two methods and/or two datasets using GAPIT version 3, mrMLM 4.0 and FarmCPU; or sharing the same haploblock to the markers detected in GModel2.

Trait	SNP	Chr	Pos	Detected dataset	Detected model	Marker effect	r2 (%)
NW	Peach_AO_0100564	1	33.21	2016; Ave	G model	−1.28∼−2.54	NA
	Peach_AO_0243498	2	12.10	2015	SUPER; Blink; pLARmEB; ISIS EM-BLASSO	2.98	15.53∼17.90
	Peach_AO_0419022	4	2.96	Ave	FASTmrMLM; ISIS EM_BLASSO	1.96∼2.21	15.24∼15.63
	SNP_IGA_386560	4	4.20	Ave	FASTmrMLM; pLARmEB; ISIS EM_BLASSO	1.39∼1.50	11.39∼17.38
	**Peach_AO_0576871**	5	11.28	Ave	G model	0.65	NA
	**Peach_AO_0577125**	5	11.35	2016	G model	−1.11	NA
	Peach_AO_0602163	6	3.76	Ave	MrMLM; FASTmrMLM; FASTmrEMMA; pKWmEB; G model	0.0001∼ 1.12	0∼22.76
	SNP_IGA_695629	6	28.36	2016; Ave	pLARmEB; G model	−1.21∼0.56	14.52
	Peach_AO_0766421	7	17.96	Ave	mrMLM; pKWmEB	1.28∼3.12	21.16
	Peach_AO_0860807	8	17.74	2016	FASTmrEMMA; pKWmEB; ISIS EM-BLASSO	−2.95∼-2.66	0∼18.19
W	Peach_AO_0452353	4	13.12	2015; Ave	MLMM; pLARmEB	−6.52∼-5.92	31.44∼40.45
	Peach_AO_0692414	6	30.69	2016; Ave	FarmCPU; FASTmrMLM; pLARmEB; ISIS EM-BLASSO	−4.54∼-3.40	4.82∼17.43
	Peach_AO_0711526	7	3.96	2016; Ave	FASTmrEMMA; pLARmEB; ISIS EM-BLASSO	4.38∼6.68	7.54∼11.72
	SNP_IGA_733833	7	5.65	2016	MLMM; FarmCPU	−7.91	NA

*Bolded markers were located within the same haploblock and detected by G model for different datasets. ^1^Physical positions of SNP markers according to Prunus persica Whole Genome v2.0 Assembly and Annotation v2.1 (GDR: https://www.rosaceae.org). ^2^‘Ave’ dataset included the individuals phenotyped in both 2015 and 2016 using the mean DSI. ^3^Chr-chromosome. ^4^Pos-position.*

### Candidate Gene Analysis

Thirteen haploblocks (H) containing markers significantly associated with brown rot DSI in peach were detected in the peach genome (Table 4). A total of 9 H were associated with brown rot inoculation responses in non-wounded fruits across the whole genome except for Chr 3, while 4 haploblocks on 3 chromosomes (Chr 4, 6, 7) were associated with brown rot inoculation responses in wounded fruits. The H5_1, on Chr 5, encompassed two markers Peach_AO_0576871 and Peach_AO_0577125 associated with NW fruit tolerance in the Ave and 2016 datasets, respectively.

**TABLE 4:** Haploblocks encompassing markers significantly associated with brown rot response in peach fruit skin and flesh.

Treatment	Hap	Chr	Start(Mb)	End (Mb)	Significant markers
NW	H1_1	1	32.84	33.32	Peach_AO_0100564
	H1_2	2	12.10	12.11	Peach_AO_0243498
	H4_1	4	2.70	2.90	Peach_AO_0419022
	H4_2	4	4.08	4.34	SNP_IGA_386560
	H5_1	5	11.28	11.62	Peach_AO_0576871, Peach_AO_0577125
	H6_1	6	2.95	3.95	Peach_AO_0602163
	H6_2	6	28.32	28.42	SNP_IGA_695629
	H7_3	7	17.96	17.99	Peach_AO_0766421
	H8_1	8	17.10	17.99	Peach_AO_0860807
W	H4_3	4	13.03	13.12	Peach_AO_0452353
	H6_3	6	29.80	30.69	Peach_AO_0692414
	H7_1	7	3.12	4.12	Peach_AO_0711526
	H7_2	7	5.53	6.34	SNP_IGA_733833

*NW, non-wounded, skin response; W, wounded, flesh response; Hap, haploblock; Chr, Chromosome; Mb, Million base.*

Candidate gene analyses using 13 haploblocks encompassing significantly associated SNPs with brown rot response in peach fruit revealed 146 predicted genes associated with pathogen defense/response in 12 haploblocks across the whole genome (Supplementary Table 3). No candidate genes were detected in H1_2. None of the significant markers were located within the detected candidate genes. In total, 97 candidate genes were detected within the haploblocks associated with skin tolerance on all chromosomes, except for Chr 3, and 49 candidate genes were located within the haploblocks associated with flesh tolerance on Chr 4, 6, 7.

Functional annotations of the predicted genes revealed genes involved in plant-pathogen interactions. In total, 56 different functional annotations were shared by the 146 predicted genes (Supplementary Table 3). Gene ontology (GO) analysis of the predicted genes revealed 44 of them having protein/DNA binding activity, such as genes annotated to leucine rich repeat (Leu-rich_rpt) and NB-ARC domain protein (NB-ARC). Fourteen predicted genes were characterized as defense response or response to stress, such as genes annotated to peroxidase and antifungal protein ginkbilobin-2 (GNK2); and 3 predicted genes were identified as being involved in cell wall modification, such as pectinesterase inhibition domain (Pectinesterase_inhib_dom) and expansin. Whereas 38 of them had unknown GO term.

To further confirm our findings, the coding sequences of the 146 predicted genes from the *Prunus persica* Whole Genome v2.0^3^ were compared to peach in NCBI nr dataset, and 25 predicted genes annotated as disease related proteins (associated with nine significant markers) were detected (Table 5). Among those genes, 16 were associated with markers detected in NW treatment on Chr 4, 5, 6, and 8, and nine of them were associated with markers detected in W treatment on Chr 6 and 7.

**TABLE 5:** Peach genes detected in NCBI nr.

Treat	Hap.	Associated marker	GDR predictied gene	NCBI nr predicted gene	Functional annotation	Identity (Evalue)
NW	4_2	SNP_IGA_386560	Prupe.4G085500	LOC18778857	pentatricopeptide repeat-containing protein At1g09900	1(0)
			Prupe.4G087700	LOC18778451	F-box protein SKIP19	1(0)
			Prupe.4G087800	LOC18778305	F-box protein SKIP19	1(0)
	5_1	Peach_AO_0576871, Peach_AO_0577125	Prupe.5G111100	LOC18777850	disease resistance response protein 206	1(0)
			**Prupe.5G111200**	LOC18776480	putative disease resistance protein RGA3	1(0)
			**Prupe.5G111300**	LOC18777168	disease resistance response protein 206	1(0)
			Prupe.5G111400	LOC18776498	dirigent protein 5	1(0)
			Prupe.5G111500	LOC18777653	disease resistance response protein 206	1(0)
	6_1	Peach_AO_0602163	**Prupe.6G039100**	LOC18770889	receptor-like protein kinase 5	0.95(0)
			**Prupe.6G039700**	LOC18772665	pathogenesis-related genes transcriptional activator PTI6	1(0)
			Prupe.6G044500	LOC18775415	peroxidase P7	1(0)
			Prupe.6G046300	LOC18773643	wound-induced protein 1	1(0)
	6_2	SNP_IGA_695629	Prupe.6G319800	LOC18774388	F-box/kelch-repeat protein At3g06240	1(0)
	8_1	Peach_AO_0860807	Prupe.8G173200	LOC18768962	dehydrin ERD10	1(0)
			**Prupe.8G173600**	LOC18768999	TMV resistance protein N	1(0)
			**Prupe.8G173700**	LOC18768999	TMV resistance protein N	1(0)
W	6_3	Peach_AO_0692414	Prupe.6G352100	LOC18775270	leucine-rich repeat receptor-like protein kinase PXL1	1(0)
			Prupe.6G352900	LOC18772881	rust resistance kinase Lr10	1(0)
			Prupe.6G354000	LOC18773984	dehydration-responsive element-binding protein 2D	1(0)
			Prupe.6G354900	LOC18775269	probable leucine-rich repeat receptor-like protein kinase At5g63930	0.99(0)
			Prupe.6G355300	LOC18775256	pentatricopeptide repeat-containing protein At5g18390	1(0)
			**Prupe.6G357200**	LOC18775405	wall-associated receptor kinase-like 1	1(0)
			**Prupe.6G359800**	LOC18774361	leucine-rich repeat receptor-like protein CLAVATA2	1(0)
	7_2	SNP_IGA_733833	Prupe.7G032500	LOC18770698	receptor-like protein kinase 2	0.91(0)
		SNP_IGA_733833	Prupe.7G032800	LOC18770889	receptor-like protein kinase 5	0.92(0)

*Treat, treatment; Hap, haploblock; NW, non-wounded, skin response; W, wounded, flesh response. Bolded, candidate genes detected in Balsells-Llauradó et al. (2020).*

## Discussion

### Phenotypic Variation for Brown Rot Tolerance

Different phenotypic responses between NW and W treatments observed in this study confirmed previously suggested role of fruit epicarp as an important resistance barrier against *Monilinia* spp. infection (Gradziel and Wang, 1993; Gradziel et al., 2003; Martínez-García et al., 2013; Pacheco et al., 2014; Baró-Montel et al., 2019). In comparison to results obtained by Pacheco et al. (2014) and Baró-Montel et al. (2019), the DSI obtained in wounded and non-wounded fruits were relatively low. This could be due to the different phenotyping techniques and *Monilinia* spp. strains used in these previous studies. Pacheco et al. (2014) and Baró-Montel et al. (2019) incubated the inoculated fruits for 5 days before recording the phenotypic data, while we incubated our material for 3 days. In addition, the inoculum concentrations used in their studies were higher in comparison to the concentrations used in the present work. The DSI identified in this work, revealed similar patterns to the ones observed by Martínez-García et al. (2013). Significant correlations between NW and W treatment were detected within the year in both experimental years (Table 1), suggesting same genetic component might be involved in both skin and flesh tolerance to brown rot in peach. The correlations between NW and W DSI observed in our study are in agreement with the results reported by Pacheco et al. (2014) in peach and Walter et al. (2004) in apricot but were in contrast with Pascal et al. (1994) study in several *Prunus* cultivars. The observed discrepancy might be due to the differences in the inoculation technique as well as the genetics of both hosts and pathogen (Walter et al., 2004). Considering the correlations for NW and W DSI in two different years, W DSI showed higher correlation than NW DSI (Table 1), suggesting seasonal influence might have higher effect on skin than flesh tolerance. Disease assay used in this study was designed to represent fruit responses to brown rot infection under uniform humidity, temperature, fruit ripening stage, inoculum strain and concentration. The observed phenotypic variation of brown rot tolerance across years can be due to field environmental factors influencing structure and phytochemical composition of peach fruits. In addition, differences in inoculum application in NW assay, droplet of inoculum staying on the skin vs sliding down due to fruit size or shape, or placement, might have caused the higher DSI values in second experimental year. The estimated broad sense heritability of brown rot tolerance for both skin and flesh in our study were low comparing to the Pacheco et al. (2014). However, the estimation provided by Pacheco et al. (2014) was based on a bi-parental population with brown rot tolerance performance having interactions with maturity date.

### Genome Wide Association Study

Mechanisms of brown rot tolerance/resistance in peach fruit, despite being well researched, are still poorly understood. Previous studies revealed quantitative nature of brown rot resistance in peach influenced by different mechanical and phytochemical compound factors within the fruit epidermis and mesocarp (Gradziel and Wang, 1993; Gradziel et al., 1997, 2003; Villarino et al., 2011; de Oliveira Lino et al., 2015; Bostock et al., 1999). Recent mapping efforts used inter- (Martínez-García et al., 2013) and intraspecific (Pacheco et al., 2014) segregating populations that limited discovery to recombination present only in four individuals, one of which being almond. In this study, we report for the first time a GWAS for brown rot resistance/tolerance in peach. We successfully performed GWAS using multiple single- and multi-locus GWAS simultaneously. Reliable marker-trait association analysis revealed 4 SNPs associated with flesh and 10 SNPs associated with skin tolerance across peach genome confirming that brown rot tolerance in peach fruit is a quantitative trait under polygenic control.

None of the markers detected in our study were located within the brown rot tolerance/resistance regions reported by Martínez-García et al. (2013) and Pacheco et al. (2014). A possible explanation could be the different sources of brown rot resistance, genotyping platforms and the approach used for detecting QTLs. In this work, we used peach intraspecific material with the Brazilian landrace ‘Bolinha’ as a source of resistance, the individuals were genotyped with peach 9+9K peach SNP array (Gasic et al., 2019) and significant associations were detected using GWAS. Martínez-García et al. (2013) applied bi-parental mapping on F1 interspecific material with almond as a source of resistance and the genotyping was performed using GoldenGate platform, which is less commonly used in the peach community. On the other hand, Pacheco et al. (2014), also used bi-parental mapping approach with interspecific peach F2 material with brown rot tolerance from peach cultivar Contender, and SSR markers and only 26 SNPs from 9K SNP array v1 (Verde et al., 2012) were used for genotyping. Furthermore, under phenotyping protocol described in Pacheco et al. (2014), the disease incidence (percentage of infected fruits) in non-wounded and average lesion diameter in wounded assay were analyzed using *M. fructigena* inoculum. To our knowledge, this is the first study using GWAS to understand brown rot resistance/tolerance in peach. In both cited studies, QTLs were mapped via linkage mapping using biparental population. In our study, GWAS is applied to a broader germplasm panel compared to bi-parental linkage mapping and consider not only the recombination events that occur in a single cross, but also the historical recombination (Zhu et al., 2008) and thereby can achieve higher resolution mapping.

Two associated markers, Peach_AO_0464476 and Peach_AO_0711526, reported in our study, were located near the 2 QTL regions on Chr 4 and Chr 7 detected by Baró-Montel et al. (2019) in a BC1 interspecific population between almond ‘Texas’ and peach ‘Earlygold’ (TE1). According to the haploblock analysis carried out in our GWAS panel, the two nearest markers, SNP_IGA_440110 (Chr 4-16,076,720) and SNP_IGA_781455 (Chr 7-16,567,648) reported by the cited authors, were located within the same haploblock as Peach_AO_0464476 (Chr 4-15,871,086) and Peach_AO_0771526 (Chr 7-15,932,502), respectively. However, we did not consider these two markers as reliable QTNs because they were only detected by one method and single dataset. Low overlap between our results and those reported by Baró-Montel et al. (2019) might be explained by the different genetic background of material used, type and number of markers included, and difference in phenotyping techniques. Baró-Montel et al. (2019) applied QTL mapping on an interspecific BC1 population with almond as source of tolerance, used 113 SSRs and 1919 SNPs from 9K SNP array v1 (Verde et al., 2012) for genotyping, and analyzed lesion diameter and disease incidence. In our study, the tolerance came from Brazilian landrace ‘Bolinha’, we used improved peach 9+9K SNP array (Gasic et al., 2019), where newly added SNPs, labeled as “Peach_AO”, are not present on 9K SNP array v1 (Verde et al., 2012), and recorded phenotypic differences as disease severity index.

Comparison between the associated markers identified by GModel2 and other multi-locus methods, revealed two markers, Peach_AO_0602163 and SNP_IGA_695629, detected by both approaches. Since different marker sets were used in GModel2 and FarmCPU analysis, low number of common detected markers was expected. Comparing the models used in this study, multi-locus models showed significantly higher detection power then the single-locus models. Only 2 markers were detected using single-locus models (MLM and SUPER) which might be explained by the polygenic background of brown rot tolerance. It has been suggested that the stringent criteria used for detecting QTNs in a single-locus models might be too conservative for traits controlled by multiple genes with small effect (Wang et al., 2016) and implementation of multi-locus models was recommended. QQ plots from single-locus and multi-locus models (Supplementary Figures 2, 4, 5), revealed MLM (Ave_W), MLMM (Ave_NW) and FarmCPU (2016_W) models being fitted properly to the datasets. By conducting GWAS with multiple methods, we narrowed down list of significant markers to 14, in which 5 were detected in different datasets with same/different models, and 2 with more than 4 models with same/different datasets. The findings in our study support combining single- and multi-locus models to improve the reliability and robustness of GWAS analysis. Marker Peach_AO_0452353, associated with flesh resistance on Chr 4, explained the highest phenotypic variance (*r*^2^ = 31.44 – 40.45), with homozygous genotypes having lower DSI than heterozygous suggesting negative epistatic effect to the brown rot response.

Although this study provided useful insights into the genetics mechanisms controlling brown rot resistance/tolerance in peach, effects of the significant SNPs were small and explained a limited percentage of phenotypic variance, suggesting the necessity of further studies using additional sources of brown rot resistance/tolerance and years of phenotypic evaluations. In addition, expression studies could provide evidence of genes specifically involved in brown rot repornse, or variant. These results also suggest that genomic prediction, as alternative approach for DNA informed breeding, might be better suited considering the polygenic background and low heritability of this trait.

### Candidate Gene Analysis

Candidate gene analyses based on the 14 reliable significant QTNs reported in our study are in agreement with differentially expressed genes (DEG) reported by Balsells-Llauradó et al. (2020). Out of 146 candidate genes indicated in brown rot tolerance reported in our study (Supplementary Table 3) 58 were differentially expressed in mature fruit of nectarine cultivar Venus 48 h after inoculation with *M. laxa* (Balsells-Llauradó et al., 2020). Moreover, out of 25 candidate genes annotated in disease resistance response in our study (Table 5), 8 were also reported by Balsells-Llauradó et al. (2020) in mature fruit.

**FIGURE 4:**
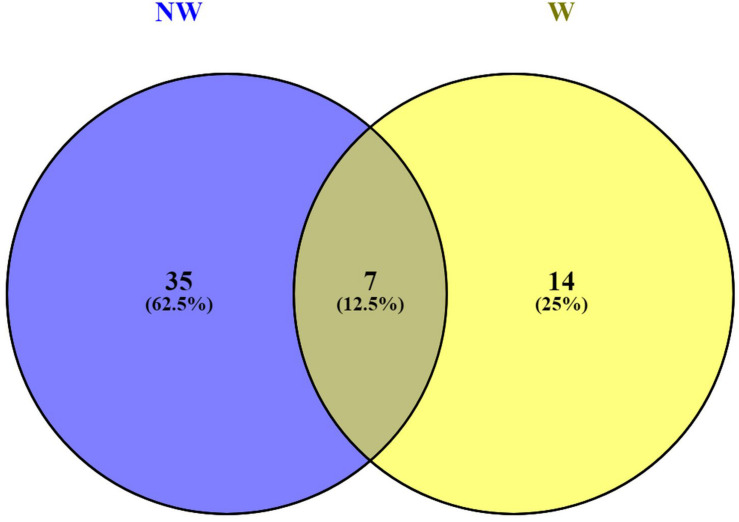
Venn diagram for functional annotations shared by candidate genes associated with non-wounded (NW) and wounded (W) peach tolerance to brown rot infection.

Functional annotations of the 146 predicted genes in *Prunus persica* Whole Genome v2.0^2^ (Supplementary Table 3) associated with peach fruit response to brown rot infection revealed plant-pathogen interactions. Functional annotations for candidate genes associated with NW and W DSI, revealed 7 annotation terms, including leucine-rich repeat protein (LRR) (Leu-rich_rpt), shared by both traits (Figure 4). LRRs are short sequence motifs present in NB-LRR, which is the largest class of plant resistance genes (R genes) (Kobe and Kajava, 2001; Dangl and Jones, 2001). LRR receptor-like protein kinase has been suggested to have an important role in signaling during the pathogen recognition (Romeis, 2001; Afzal et al., 2007).

Annotations related to cell wall modification (Expansin and Pectinesterase_inhib_dom) were only detected with the candidate genes associated with NW DSI, suggesting the importance of mechanical defense mechanism to the skin resistance to brown rot (Gradziel et al., 2003; de Oliveira Lino et al., 2015).

After comparison to the NCBInr database, the candidate gene list was narrowed down to 25 (Table 4). Eight genes detected on Chr 5, 6 and 8 (*Prupe.5G111100, Prupe.5G111200, Prupe.5G111300, Prupe.5G111400, Prupe.5G111500, Prupe.6G352900, Prupe.8G173600, Prupe.8G173700*,) were associated with disease resistance. Four predicted genes on chromosome 5 (*Prupe.5G111100*, *Prupe.5G111200*, *Prupe.5G111300*, *Prupe.5G111500*) are annotated as disease resistance response proteins. Although the specific function of these disease resistance proteins is still unclear, previous studies suggested the presence of series of LRRs that can interact with a pathogen avirulent gene and trigger the cell defense response including cell death (Dangl and Jones, 2001; Kobe and Kajava, 2001; Belkhadir et al., 2004). Additionally, *Prupe.5G111400* is annotated as dirigent protein (DIR) 5. The DIRs are important in lignan and lignin biosynthesis and can contribute to plant defense against pathogens (Paniagua et al., 2017). Lignans can inhibit microbe-derived degradative enzymes (polygalacturonases, cellulases and glucosidases), and lignins can stabilize the cell wall and form a barrier against microbial pathogen to limit the spread of pathogen derived toxins and enzymes (Grabber, 2005; Paniagua et al., 2017). Two candidate genes detected on Chr 8 were annotated as TMV resistance protein. This gene encodes for a TIR-NB-LRR protein (Whitham et al., 1994) and can recognize a TMV elicitor p50 and induce defensive response (Taku et al., 2018). Genes encoding TMV resistance protein were also reported by Martínez-García et al. (2013) to be involved in brown rot resistance in peach. Another candidate gene on Chr 6, annotated as rust resistance kinase Lr10 in wheat (Feuillet et al., 1997), encodes a receptor-like protein kinase.

*Prupe.6G039700*, *Prupe.6G044500*, and *Prupe.6G046300*, are related to pathogenesis-related genes transcriptional activator PTI6 (Pti6), peroxidase P7 and wound-induced protein 1 (Wun1), respectively. In tomato, Pti6 is a transcription factor that can interact with the product of Pto disease resistance gene and activate defense responses. In Arabidopsis, it can activate the ethylene/jasmonic acid-response genes and salicylic acid-regulated genes that encode for the important signaling molecules in defense pathways (Gu et al., 2002). On the other hand, peroxidases are enzymes involved in cell wall polysaccharide processes (phenols oxidization, suberization, and lignification of host plant cells) during the defense reaction against a pathogen (Chittoor et al., 1999). In harvested peaches, Liu et al. (2005) detected that enhanced peroxidase activity was related to increased pathogen defense, and the same was also suggested in peach resistance to brown rot study reported by Ma et al. (2013). In potato, Wun1, encodes protein that is rapidly accumulated in response to wounding (Logemann and Schell, 1989) and is potentially related to cell death.

The wall-associated receptor kinase (WAKL) genes, that encode functional protein kinases associated with cell wall (Verica and He, 2002), was associated with brown rot infection in wounded fruits. The function of WAKL genes is not well-understood, however, studies suggested that these genes may have a role in disease resistance (Verica and He, 2002; Zhang et al., 2005; Kohorn and Kohorn, 2012).

## Conclusion

Brown rot tolerance in peach is a complex trait controlled by multiple genes. In this study, we successfully performed GWAS using both single- and multi-locus methods. A total of 14 reliable makers associated with brown rot DSI under wounded/non-wounded treatment were detected in at least two seasons and/or two different methods. Using the available genetic databases and peach genome annotation, we were able to detect a list of functional candidate genes that could provide a better understanding of the genetics mechanism that control brown rot tolerance/resistance in peach. Candidate gene analysis revealed 25 predicted peach genes associated with brown rot tolerance in wounded or non-wounded peach. This is the first report of GWAS for brown rot tolerance in peach and the information reported in this study provides foundation for genome-based DNA informed breeding for brown rot tolerance in peach.

## Data Availability Statement

The original contributions presented in the study are included in the article/Supplementary Materials, further inquiries can be directed to the corresponding author/s.

## Author Contributions

WF did the data collection, formal analysis and drafting of the manuscript. CDSL assisted with data analysis, reviewed and edited the manuscript. KG did the conceptualization, funding acquisition, resources, supervision, reviewed and edited the manuscript. All authors read and approved the final manuscript.

## Conflict of Interest

The authors declare that the research was conducted in the absence of any commercial or financial relationships that could be construed as a potential conflict of interest.

## Footnotes

1 http://bernardo-group.org/books-and-software/

2 https://blast.ncbi.nlm.nih.gov

3 https://www.rosaceae.org
