# Supplementary figures and images for: Genome-Wide Association Study of Brown Rot (Monilinia spp.) Tolerance in Peach
Source: Front Plant Sci. 2021 Mar 9;12:635914. doi: 10.3389/fpls.2021.635914 (PMC8006439; doi:10.3389/fpls.2021.635914)

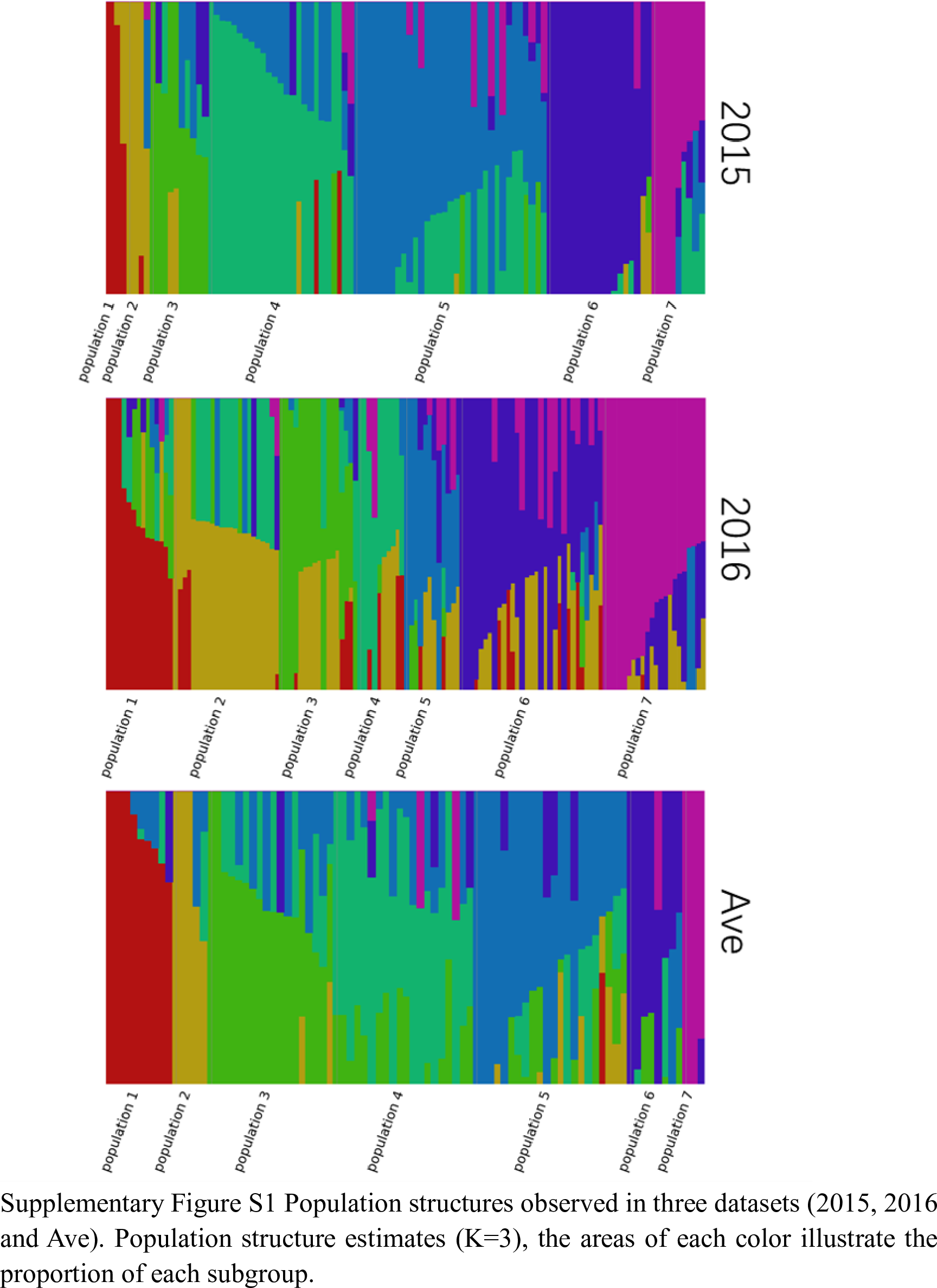

Supplement: Supplementary file 1 [file Image_1.tif]

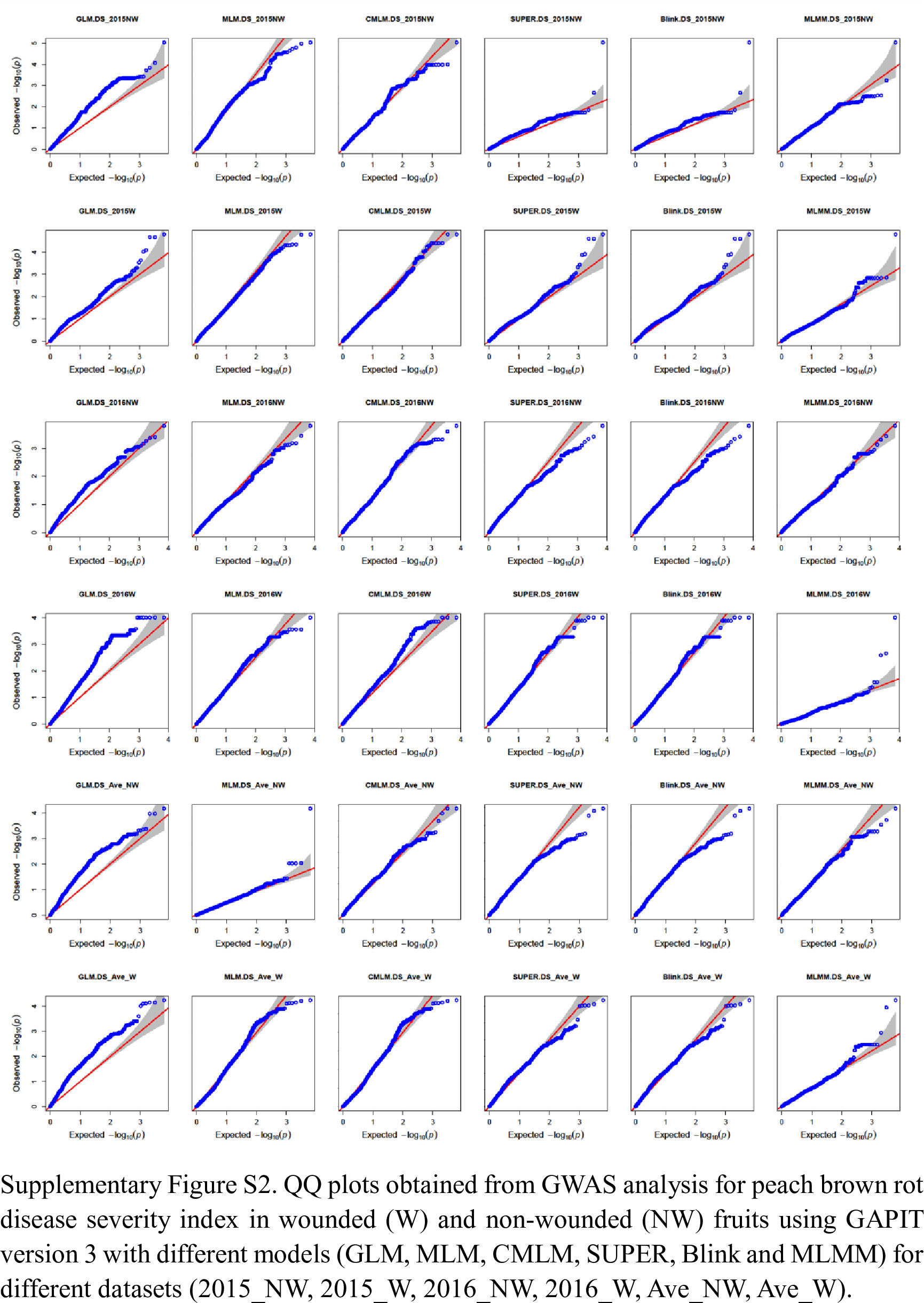

Supplement: Supplementary file 2 [file Image_2.tif]

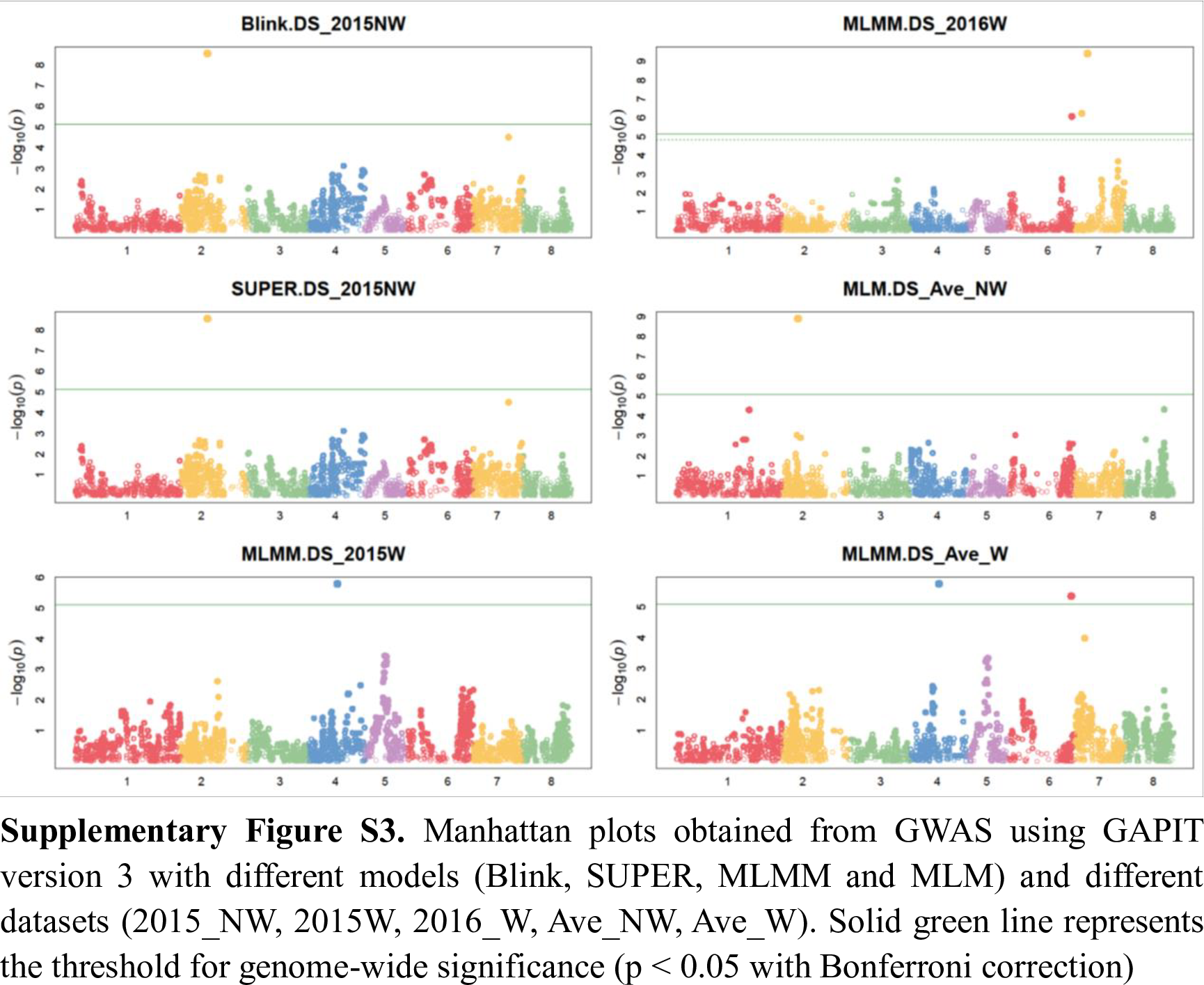

Supplement: Supplementary file 3 [file Image_3.tif]

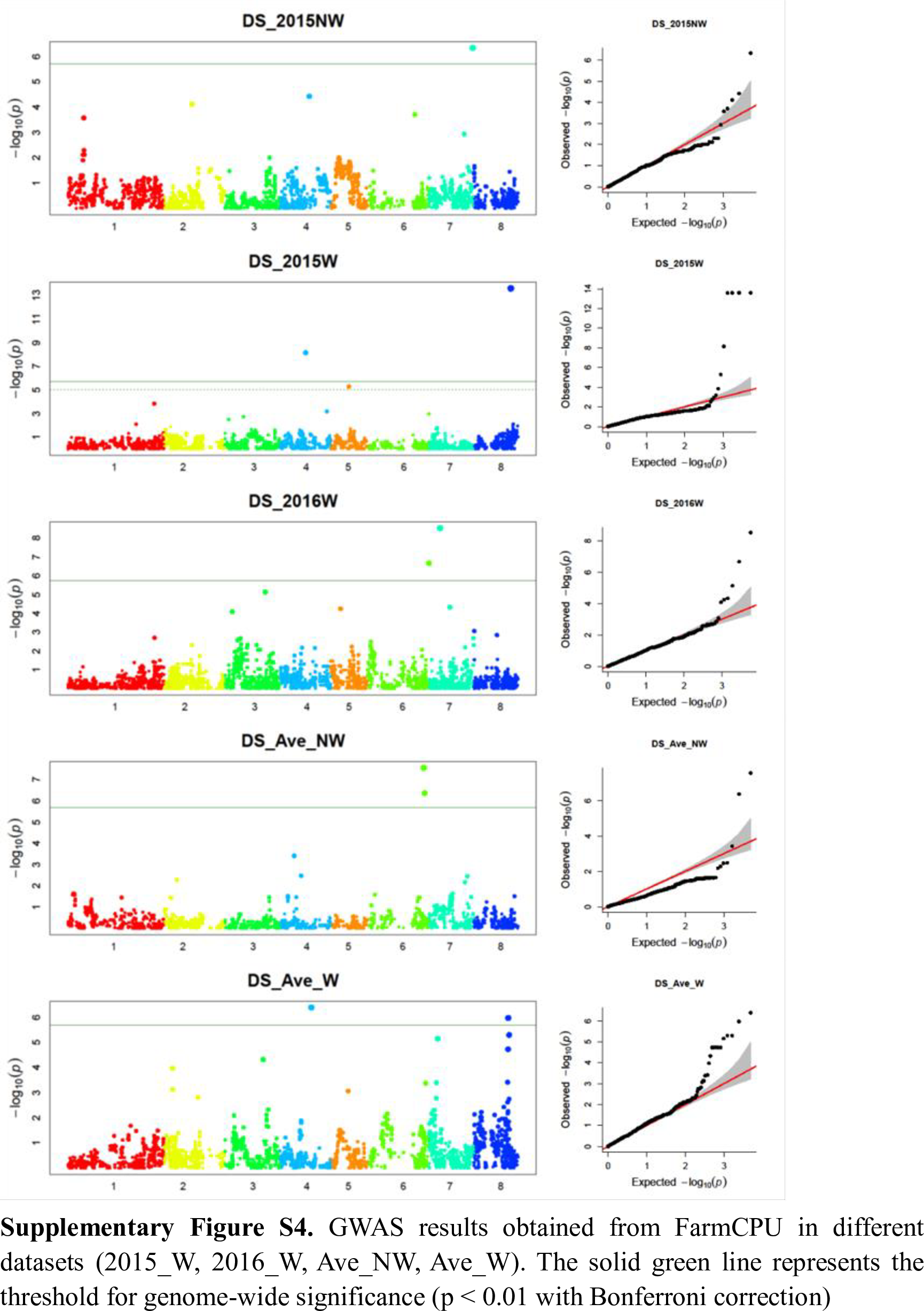

Supplement: Supplementary file 4 [file Image_4.tif]

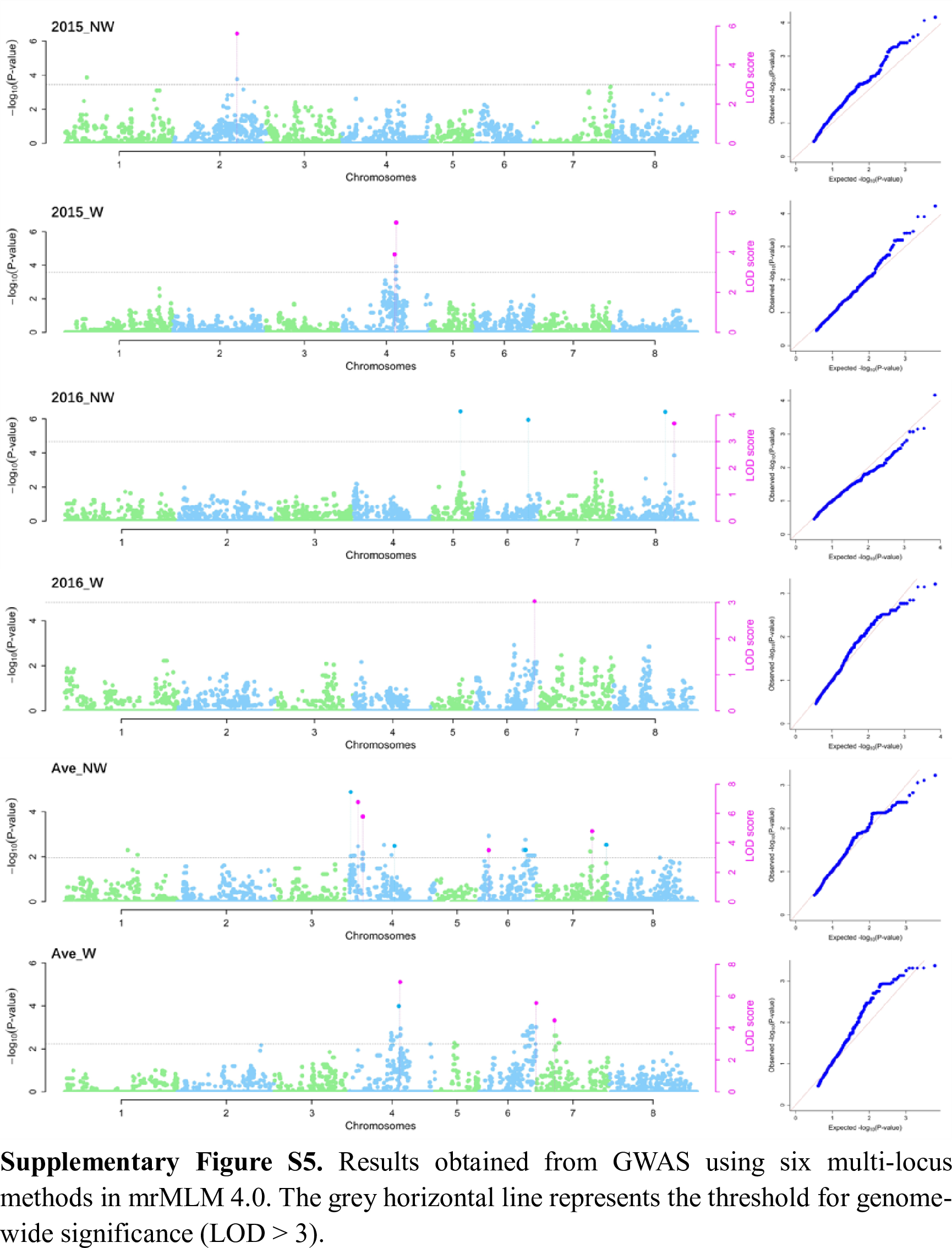

Supplement: Supplementary file 5 [file Image_5.tif]
